# Elevated circulating dipeptidyl peptidase 3 (cDPP3) as a predictor of postoperative complications in esophagectomy patients

**DOI:** 10.1007/s00423-025-03893-4

**Published:** 2025-10-20

**Authors:** Valerie Isabel Nottberg, Christine Marie Torrey, Courtney Metz, Natasha Irene Schweitzer, Simone Schewe, Mara R. Goetz, Matthias Reeh, Jakob R. Izbicki, Jan Bardenhagen, Asmus Heumann

**Affiliations:** 1 Department of General, Visceral and Thoracic Surgery, University Medical Center Hamburg-Eppendorf, Martinistraße 52, 20246 Hamburg, Germany; 2 Department of General, Visceral and Thoracic Surgery, Kath. Marienkrankenhaus Hamburg, Alfredstraße 9, 22087 Hamburg, Germany

**Keywords:** Biomarker, cDPP3, Esophagectomy, Sepsis, Anastomotic insufficiency, Postoperative complications

## Abstract

**Purpose:**

Esophagectomy for esophageal cancer carries high postoperative morbidity and mortality. Circulating dipeptidyl peptidase 3 (cDPP3) is a novel biomarker linked to organ failure in acute care. This study evaluates its prognostic value for predicting organ dysfunction and mortality post-esophagectomy.

**Methods:**

This prospective, single-center study included 20 esophagectomy patients. cDPP3 levels were measured preoperatively and at 24, 72, 120, and 168 h postoperatively using a Nexus IB10 Analyzer. Postoperative complications, including pneumonia, anastomotic insufficiency, and SOFA score-based organ dysfunction, were recorded. Correlation analysis assessed associations between cDPP3 levels and clinical outcomes.

**Results:**

Among 20 patients, the most common complications were pneumonia (*n* = 12), sepsis (*n* = 17), and anastomotic insufficiency (*n* = 5). Elevated cDPP3 levels correlated with SOFA score increases ≥ 2 (*p* < 0.01) and severe complications, including prolonged ICU stays. cDPP3 levels at 72 h were significantly associated with anastomotic insufficiency (*p* < 0.01). A negative correlation was observed between intraoperative volume and cDPP3 levels at 24 h (*r* = -0.495, *p* = 0.03).

**Conclusions:**

cDPP3 is a potential biomarker for postoperative complications after esophagectomy. Its elevation correlates with increased morbidity, highlighting the need for vigilant perioperative management.

**Supplementary Information:**

The online version contains supplementary material available at 10.1007/s00423-025-03893-4.

## Introduction

Esophageal cancer is often diagnosed at advanced stages due to subtle symptoms and rapid progression. Esophagectomy remains a key treatment for localized disease but carries high morbidity and mortality [1, 2]. Regardless of the surgical approach, it induces significant operative stress and ischemia-reperfusion injury, triggering systemic inflammation. Complications such as organ failure, shock, and sepsis increase mortality [3]. Septic events occur in ~ 25% of cases, reducing short-term survival and increasing the need for cardiovascular support, mechanical ventilation, and renal replacement therapy [4]. Cardiovascular complications affect ~ 33% of esophagectomy patients, leading to severe outcomes like myocardial infarction and heart failure [5]. Single-lung ventilation raises pneumonia risk (10–20%), impacting long-term survival [6]. Anastomotic leaks (10–25%) prolong recovery and lower five-year survival [7]. Proper fluid management is crucial, as intraoperative volume overload heightens respiratory and anastomotic risks [8]. Multidisciplinary monitoring and timely intervention are essential to improve outcomes.

Circulating dipeptidyl peptidase 3 (cDPP3), a zinc-dependent metallopeptidase, degrades bioactive peptides like angiotensin II [9]. It is a promising biomarker for predicting adverse outcomes in critically ill patients, including in-hospital mortality and postoperative complications [10]. Elevated cDPP3 levels correlate with hemodynamic instability and poor outcomes [11, 12]. By contributing to vasodilation and angiotensin II degradation, cDPP3 may promote circulatory failure [13]. Elevated perioperative cDPP3 could serve as an early indicator of severe complications, enabling targeted interventions to reduce esophagectomy-related risks.

This study evaluates cDPP3 as a predictor of severe postoperative complications, including organ dysfunction and mortality. By integrating cDPP3 measurement into perioperative management, we aim to validate its role as a biomarker for sepsis and other postoperative risks.

## Outcome

The primary outcome of this study was to assess the feasibility of cDPP3 blood analysis in esophagectomy patients and its correlation with negative postoperative outcomes, including anastomotic insufficiency, sepsis, pneumonia, acute kidney injury, and cardiac arrhythmia.

## Methods

### Patients and study design

This prospective, single-center study was conducted at University Clinic-Hamburg Eppendorf, Germany, within a biobank study framework. Approved by the Hamburg Ethics Committee (PV3548), it adhered to the Declaration of Helsinki. Participants were consenting adults (≥ 18 years) undergoing esophagectomy for esophageal tumors between April and November 2022.

Exclusion criteria included metastatic malignancy, corticosteroid or immunosuppressant use within four weeks, severe systemic disease with a life expectancy under six weeks, immune compromise (e.g., HIV), preoperative dialysis, septic shock, vasopressor use, or pregnancy (see Table 1). Among 25 screened patients, 20 met inclusion criteria. Five were excluded: one due to palliative surgery and four due to extended disease discovered intraoperatively, preventing esophagectomy. Recruitment occurred during surgical consultation.

**Table 1:** Inclusion and exclusion criteria

	Inclusion criteria			Exclusion criteria	
Patients age ≥ 18 years			Corticosteroids or immunosuppressive agents within four weeks preceding surgery		
Ability to provide written and oral informed consent			Immunosuppression or immune deficiency disorders, severe systemic illness (life expectancy less than 6 weeks		
Presence of esophageal cancer with need for surgery • Adenocarcinoma • Squamous cell carcinoma ESCC • Neuroendocrine carcinoma • Gastrointestinal stromal tumor			Preexisting indication for renal dialysis Preoperative septic shock or the necessity for vasopressor support Pregnancy		
Undergoing esophagectomy • Da Vinci assisted • Open • Laparoscopic			Individuals currently incarcerated or institutionalized		

### Data collection and clinical assessment

At intake, detailed medical histories were reviewed to assess study eligibility. Demographic data (gender, age), vital signs, pre-existing conditions, current diagnoses, and laboratory parameters were recorded. Operation duration and intra-/postoperative volume management were documented to analyze their impact on outcomes and complication rates.

Following esophagectomy, patients underwent daily clinical assessments for 30 days or until hospital discharge. Wound conditions were monitored, and complications such as acute kidney injury requiring dialysis, cardiogenic shock, and respiratory failure needing invasive ventilation were documented. The SOFA score assessed sepsis onset, defined by a two-point increase [14]. Additionally, necessary postoperative interventions (surgical, endoscopic, or radiological) were recorded. Surgical complications were classified using the Clavien-Dindo system and the Comprehensive Complication Index (CCI).

A total of 100 blood samples were collected from 20 patients at five pre- and postoperative time points: once before surgery (up to 24 h prior) and at 24, 72, 120, and 168 h postoperatively. Routine blood tests, including complete blood count (CBC), liver transaminases, kidney function, and infection/inflammation markers, were performed at the UKE in-house central laboratory.

cDPP3 levels were measured using a Nexus IB10 Analyzer point-of-care testing (POCT) device, complying with RiliBÄK guidelines (German regulatory standards for medical laboratory accuracy). The immunoassays are validated for in vitro quantitative cDPP3 determination in whole blood and plasma collected in EDTA tubes. Samples were processed immediately under normal laboratory conditions or stored at 4 °C for up to 24 h per manufacturer recommendations.

The analytical range of the Nexus IB10 analyzer for cDPP3 concentrations is 5–150 ng/ml. In this study, values ≥ 14 ng/ml (the median in healthy adults) were considered elevated, while those above 41 ng/ml were classified as high, reflecting the 97.5th percentile [12].

### Statistical analysis

A formal statistical consultation was conducted before analysis to ensure appropriate methodologies and rigorous evaluation techniques. Descriptive statistics summarized patient demographics, clinical variables, and laboratory measurements. Data are presented as means with standard deviations, medians with interquartile ranges, or counts and percentages, as appropriate. Continuous variables were assumed to follow a normal distribution.

For comparisons between two independent groups (e.g., patients with vs. without postoperative complications), the non-parametric Mann-Whitney U (MW) test was used. To assess time-dependent differences, comparisons were performed separately for each predefined postoperative timepoint (baseline, 24 h, 72 h, 120 h, 168 h). To control for multiple testing, Bonferroni correction was applied by multiplying each p-value by the number of comparisons per outcome (i.e., five timepoints). A corrected p-value < 0.05 was considered statistically significant.

Pearson correlation analyzed associations between elevated cDPP3 levels and specific complications, including pneumonia, anastomotic insufficiency, and organ dysfunction, defined as a ≥ 2-point increase in the SOFA score. Changes in cDPP3 levels over time were analyzed using repeated-measures ANOVA and the non-parametric Friedman test. Although a multivariable logistic regression analysis was initially planned to examine the combined effect of cDPP3, operative time, sepsis, and CRP levels on postoperative outcomes, it was ultimately not performed due to the limited sample size and unfavorable data distribution, which would have rendered the results statistically unreliable. Statistical analyses were performed using R software (version 4.4.2) and GraphPad Prism (version 10.2.2). All tests were two-tailed, with a significance threshold of *p* < 0.05.

## Results

### Demographics and clinical characteristics

The study cohort included 20 patients, 90% male and 10% female, aged 42 to 82 years (median age 61). Histological diagnoses comprised adenocarcinoma (*n* = 14), squamous cell carcinoma (*n* = 4), neuroendocrine carcinoma (*n* = 1), and gastrointestinal stromal tumor (*n* = 1). Esophagectomy approaches included Da Vinci-assisted thoracoabdominal (*n* = 9), Da Vinci Hybrid cervicothoracoabdominal (*n* = 1), open thoracoabdominal (*n* = 1), and laparoscopic thoracoabdominal (*n* = 9).

Neoadjuvant treatment was administered to 10 patients, while the remaining 10 underwent surgery without prior therapy. Seven patients were smokers (mean 42 pack-years), and three reported current or past alcohol use. Additional demographic and medical history details are provided in Table 2.

**Table 2:** Mean ± SD DPP3 levels by medical condition (pneumonia, cardiac arrhythmia, anastomotic insufficiency, SOFA score increase ≥ 2) over all timepoints

	Pneumonia		*p*	Cardiac arrhythmia		*p*
	Yes	No		Yes	No	
DPP3 in ng/ml	43.82$\:\pm\:$38.58	34.66$\:\pm\:$31.79	0.26	55.05$\:\pm\:$41.42	35.39$\:\pm\:$32.77	0.17
	Anastomotic insufficiency			SOFA Score increase ≥ 2		
	Yes	No		Yes	No	
DPP3 in ng/ml	56.38$\:\pm\:$47.77	35.72$\:\pm\:$32.35	0.26	43.40$\:\pm\:\:$36.67	19.90$\:\pm\:$14.78	**< 0.01**

### Postoperative complications

Major complications observed included pneumonia (*n* = 12), anastomotic insufficiency (*n* = 5), cardiac arrhythmia (*n* = 3) and sepsis (*n* = 17). Postoperative management for pneumonia included intubation and/or supplemental oxygen delivery (nasal cannula or high-flow oxygen) starting from the first postoperative day, with an average postoperative ventilation duration of 5.7 days ± 9.4. The average length of ICU stay was 9.8 days ± 10.8 postoperatively. Morbidity was assessed using the Clavien-Dindo classification; two patients experienced minor morbidity (grades I or II), fifteen had major morbidity (grades IIIa/b or IV), and three exhibited no complications (grade 0). Thirty-day mortality risk was assessed using the Comprehensive Complication Index (CCI), which had a median value of 47.2 ± 28 (Table 2). One patient died during the 30 day follow-up.

### Clinical significance of the perioperative cDPP3 levels

**Fig. 1:**
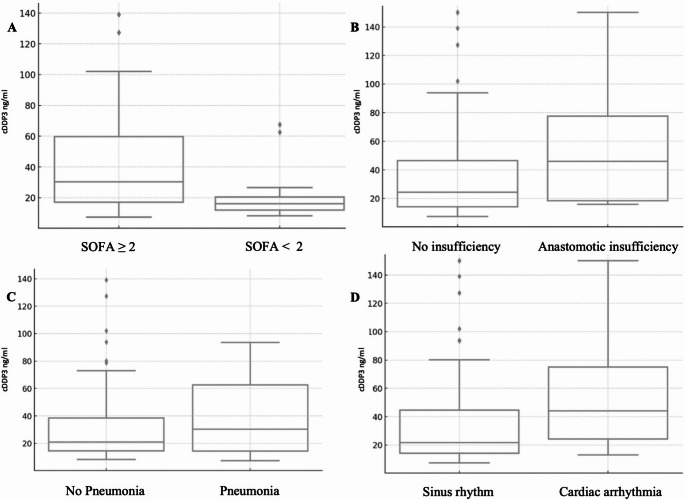
Box plots of cDPP3 distribution in patients with sepsis (**A**), anastomotic insufficiency (**B**), pneumonia (**C**), and cardiac arrhythmia (**D**)

**Fig. 2:**
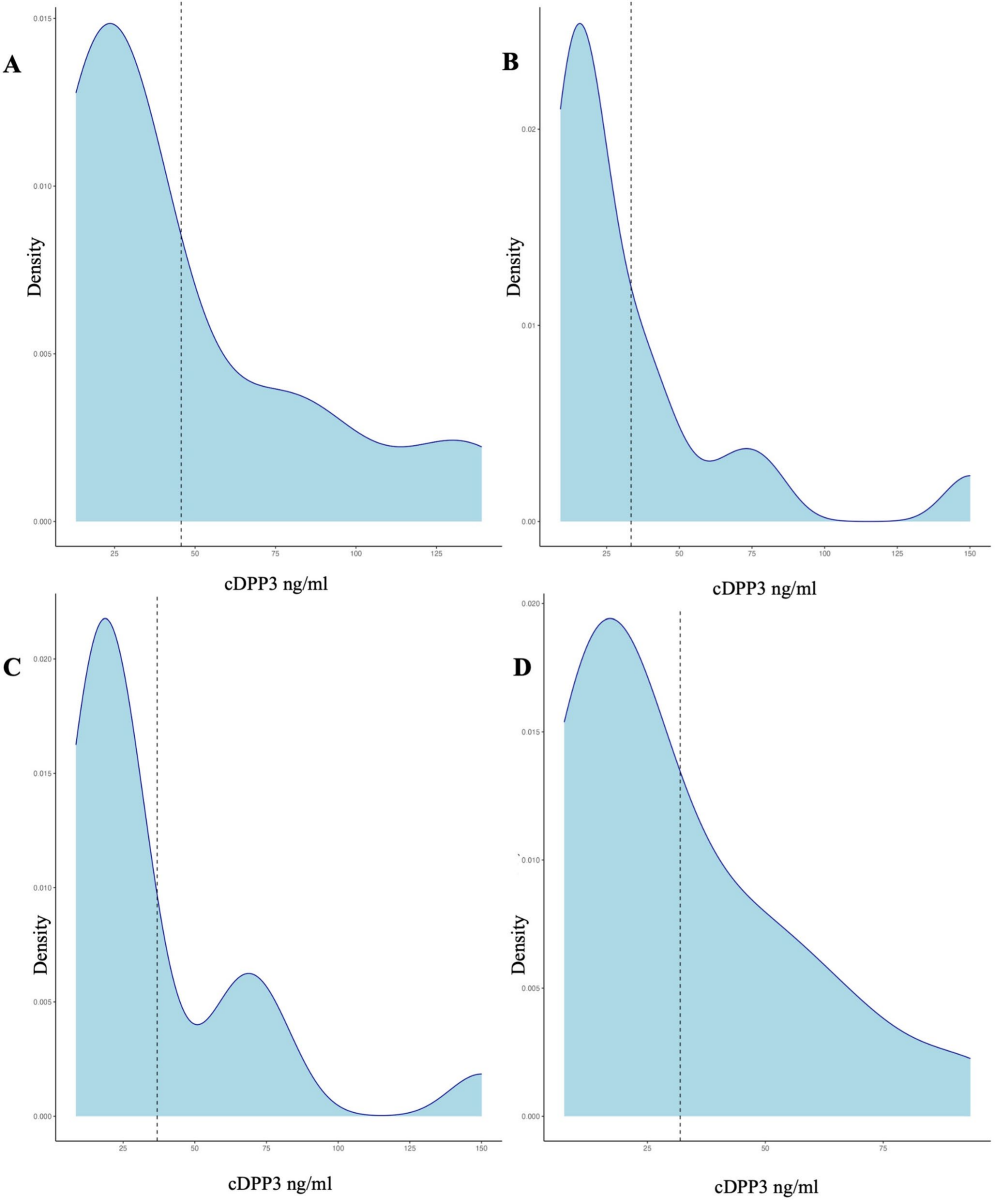
Density plots of postoperative cDDP3 distribution (ng/ml) at 24 h (**A**), 72 h (**B**), 128 h (**C**) and 160 h (**D**)

Certain complications correlated with elevated cDPP3 levels, particularly pneumonia, anastomotic insufficiencies, prolonged ICU stays, and mortality (Fig. 1). Postoperative cDPP3 levels were generally elevated, peaking on days one and five (Fig. 2). Repeated measures analysis showed significant differences in perioperative cDPP3 levels across time points (*p* = 0.02), while CRP (*p* = 0.12) and leukocyte levels (*p* = 0.74) remained unchanged. Seven patients had high baseline cDPP3 levels, all of whom developed postoperative complications, though this association was not statistically significant. High cDPP3 levels at 168 h postoperatively were significantly associated with ICU stays exceeding seven days (Pearson (r) = 0.45, *p* < 0.05). A Pearson-correlation was observed between CRP and cDPP3 at 72 h postoperatively and anastomotic insufficiency (*p* < 0.01), as well as between CRP and cDPP3 at 24 h and pneumonia (*p* < 0.05). Elevated cDPP3 levels at 72 h were also associated with cardiac arrhythmias (*p* = 0.02). Furthermore, cDPP3 demonstrated a significant correlation with SOFA score increases greater than two, beginning at 72 h postoperatively, with notable variability compared to patients without an increase at 120 and 168 h (*p* < 0.05).

cDPP3 levels differed significantly between patients with and without anastomotic insufficiency, particularly at 24 h post-surgery (Mann-Whitney U test with Bonferroni correction, *p* = 0.01), underscoring its potential as a predictive biomarker. A detailed overview of all measured parameters, timepoints, and corresponding statistical results is provided in the supplementary material.

There was a negative correlation between operation time and elevated cDPP3 levels (> 14 ng/ml) 24 h postoperative (*r* = −0.562, *p* = 0.01) detected. A significant negative correlation was also observed between intraoperative volume given and postoperatively elevated cDPP3 levels (> 14 ng/ml) on Day 1 (*r* = −0.495, *p* = 0.03).

### Predictive accuracy of cDPP3 for the development of postoperative organ dysfunction

Patients with sepsis (SOFA score ≥ 2) had significantly higher cDPP3 levels throughout all time points (*p* < 0.01, Table 3). Patients with pneumonia, anastomotic insufficiency, and cardiac arrhythmias also had higher cDPP3 levels, though these associations were not statistically significant (Fig. 1). Patients with a SOFA score ≥ 2 typically had higher levels of all markers, particularly notable in cDPP3 and CRP immediately post-operation and at later stages (Table 1).

**Table 3:** Patient characteristics, medical history, tumor entity, surgical details, and postoperative outcomes by cDPP3 classification (normal, elevated, high)

		All patients (*n* = 20)	Normal DPP3 < 14ng/ml	Elevated DPP3 > 14-41ng/ml		High DPP3 > 41ng/ml
Patient characteristics at baseline						
Sex male female		18(9%) 2 (0%)	6 (3%) 1 (0%)		5 (2%) 1 (0%)	7 (3%) 0 0%)
Mean Age (years)		61.2 ± 9.4	59 ± 9.4		64.3 ± 10.9	60.7 ± 8.7
Medical history at baseline						
Hypertension	Yes No	8 12	1 (13%) 7 (8%)		3 (38%) 5 (6%)	4 (50%) 4 (5%)
Coronary heart disease	Yes No	7 13	1 (14%) 6 (8%)		2 (29%) 5 (7%)	4 (57%) 3 (4%)
Other	Yes No	17 3	6 (35%) 11 (6%)		5 (29%) 12 (7%)	6 (35%) 11 (6%)
Neoadjuvant therapy	Yes No	10 10	6 (60%) 4 (4%)		2 (20%) 8 (8%)	2 (20%) 8 (8%)
Tumor entity at baseline						
Squamous cell carcinoma	Yes No	4 16	1 (25%) 3 (7%)		2 (50%) 2 (5%)	1 (25%) 3 (7%)
Adenocarcinoma	Yes No	14 6	6 (43%) 8 (5%)		2 (14%) 12 (8%)	6 (43%) 8 (5%)
Other (GIST, NEC)	Yes No	2 18	0 (0%) 2(10%)		2 (100%) 0 (%)	0 (0%) 2 (10%)
Surgical approach and techniques *(mean values across postoperative period)*						
Da-Vinci-assisted Esophagectomy Mean Operation time (min)	Yes No 452.5 ± 104.5	9 11	0 (0%) 9(10%)		5 (56%) 4(4%)	4 (44%) 5(5%)
Open Operation time (min)	Yes No 300	1 19	0 (0%) 1(10%)		0 (0%) 1(10%)	1 (100%) 0(%)
Laparoscopic Operation time	Yes No 436.4 ± 68.4	10 10	2 (20%) 8(8%)		7(70%) 3(3%)	1 (10%) 9(9%)
Mean Operation time (min)	Above 435.9 ± 81.4	7	0 (0%) 7(10%)		5 (71%) 2(2%)	2 (29%) 5(7%)
Perioperative fluid management *(mean values across postoperative period)*						
Mean Intraoperative Fluid Balance in ml	Above 4512.5 ± 1507.7	8	0 (0%) 8(10%)		5 (63%) 3(3%)	3 (37%) 5 (6%)
Mean Intraoperative given fluid in total in ml	Above 6412.5 ± 1914.8	6	0 (0%) 6(10%)		4 (67%) 2 (3%)	2 (33%) 4 (6%)
Mean fluid given postoperatively in ml 24 h in total 72 h in total	Above 1806.3 ± 2762.7 11838.2 ± 3200.3	13 12	1 8%) 0(0%)		9 (9%) 9 75%)	4 (1%) 3 25%)
Postoperative outcomes *(mean values across postoperative period)*						
Postoperative antibiotic therapy	Yes No	13 7	0 (0%) 13 (10%)		9 (69%) 4 (3%)	4 (31%) 9 (6%)
Anastomotic insufficiency	Yes No	5 15	0 (0%) 5 (10%)		4 (80%) 1 (2%)	1 (20%) 4 (8%)
AKIN						
I		3	0 (%)		2 (6%)	1 (3%)
II		1	0 0%)		1 (10%)	0 0%)
III		2	1 50%)		1 50%)	0(0%)
Need for Dialysis	Yes No	4 16	1 (25%) 3 (7%)		2 (50%) 2 (5%)	1 (25%) 3 (7%)
Cardiac arrhythmias	Yes No	4 16	0 (0%) 4 (10%)		2 (50%) 2 (5%)	1 (25%) 3 (7%)
Pneumonia	Yes No	11 9	3 (27%) 8 (7%)		4 (36%) 7 (6%)	4 (36%) 7 (6%)
Change in SOFA Score ≥ 2	Yes No	17 3	2 (12%) 15 (8%)		10 (59%) 7 (4%)	5 (29%) 12 (7%)
Onset of insufficiency (in days)	Above 5 ± 3.2	1	0 (%)		1 (10%)	0 (%)
Ventilation (in days) Mean	Above 5.7 ± 9.4	4	1 (2%)		2 (5%)	1 (2%)
ICU Duration (in days) Mean	Above 9.8 ± 10.8	6	0 (%)		4 (6%)	2 (3%)
Clavien-Dindo-Score						
0	3		1 (3%)		1 (3%)	1 (3%)
I	0		0 0%)		0 0%)	0 0%)
II	2		0(0%)		1 50%)	1 50%)
III	4		1(25%)0 (0%)		3(75%)	(0%)
IV	11				(64%)	(36%)
Mean CCI	Above 47.2$\:\pm\:$28	12	1 (8%)		7 (58%)	4 (33%)

## Discussion

Recent studies highlight circulating dipeptidyl peptidase 3 (cDPP3) as a critical biomarker for predicting adverse outcomes in severely ill patients, including burns, cardiogenic shock, and esophagectomy [10, 15]. Our findings align with prior research linking elevated cDPP3 levels to increased mortality, organ dysfunction, and hemodynamic instability in critically ill patients following major visceral surgery [11, 16].

In this study, we evaluated the prognostic value of cDPP3 in detecting early postoperative complications in esophagectomy patients. Elevated postoperative cDPP3 levels significantly correlated with severe complications, such as anastomotic insufficiency and organ dysfunction, measured by the SOFA score. This correlation underscores the potential of cDPP3 as a surgical biomarker for anticipating adverse outcomes, refining clinical monitoring, and proactively managing complications.

### Clinical significance of cDPP3

Elevated cDPP3 levels were associated with longer ICU stays and increased morbidity, reflecting postoperative stress and inflammation. Patients with high baseline cDPP3 experienced more severe complications, suggesting its utility for preoperative risk stratification, particularly in ERAS programs [17]. Dynamic postoperative changes in cDPP3 further highlight its potential as an early marker for complications like anastomotic insufficiency and cardiac arrhythmias.

In patients with organ dysfunction, elevated cDPP3 and CRP correlated with higher SOFA scores, indicating sepsis, with the strongest association from the first postoperative day onward. This reinforces cDPP3 as a potential marker for predicting sepsis, organ failure, and postoperative recovery.

### Implications for esophageal surgery and patient management

Despite advancements in surgical techniques, esophagectomy remains a high-risk procedure with significant complications [7]. The study data highlight ongoing challenges in managing these risks, particularly due to the high incidence of pneumonia and sepsis. The potential predictive capability of cDPP3 for these complications, especially when combined with traditional markers like CRP, supports a multidimensional approach to perioperative monitoring for improved patient outcomes.

Elevated cDPP3 levels, particularly at 168 h postoperatively, correlated with prolonged ICU stays and higher morbidity. This suggests that cDPP3 may serve as a biomarker for predicting postoperative outcomes and guiding targeted clinical interventions. Effective perioperative fluid management is critical, as current practices are linked to complications like pneumonia [8]. Additionally, the observed negative correlations between operative time, intraoperative volume, and elevated cDPP3 levels present an opportunity to refine surgical and anesthetic strategies, potentially reducing postoperative complications.

### Limitations and future research directions

This study has several limitations that must be considered when interpreting the results. First, the sample size was small (*n* = 20), which limits statistical power. As a result, subgroup analyses. such as those assessing specific complications, should be interpreted cautiously. Second, the study was conducted at a single center with a short follow-up period of 30 days, which may not capture delayed or long-term complications. Third, although a multivariable logistic regression was initially performed to evaluate the combined effects of cDPP3, ICU stay, operative time, sepsis, and CRP, the analysis was ultimately excluded due to the limited sample size, non-normal data distribution, and lack of statistical significance (pseudo R² = 0.2257, *p* = 0.195–0.962). Lastly, the observational design does not allow for causal inference, and all associations should be considered exploratory. Future studies with larger, multicenter cohorts and extended follow-up are needed to validate the predictive value of cDPP3 and to determine its potential role in clinical decision-making.

## Conclusion

This pilot study suggests that cDPP3 may serve as a promising biomarker for identifying patients at risk of severe postoperative complications following esophagectomy. Its correlation with SOFA scores and specific complications like anastomotic insufficiency and sepsis supports its integration into clinical protocols. However, given the study’s small sample size and exploratory design, these findings require confirmation in larger, multicenter trials before cDPP3 can be considered for routine clinical use in perioperative risk assessment.

## Supplementary Information

Below is the link to the electronic supplementary material.

Supplementary Material 1 (DOCX 23.7 KB)

## Data Availability

The datasets generated and analyzed during the current study are not publicly available due to patient privacy restrictions but are available from the corresponding author on reasonable request.
