# Supplementary material for: Elevated circulating dipeptidyl peptidase 3 (cDPP3) as a predictor of postoperative complications in esophagectomy patients
Source: Langenbecks Arch Surg. 2025 Oct 20;410(1):304. doi: 10.1007/s00423-025-03893-4 (PMC12537593; doi:10.1007/s00423-025-03893-4)
Supplement: Supplementary file 1 — Supplementary Material 1 (DOCX 23.7 KB) [file 423_2025_3893_MOESM1_ESM.docx]

| **Parameter** | **Timepoint of measurement** | **Patients (n=20)** | **Anastomotic insufficiency (n=5)** | **P MW** | **Pearson-**  **Correlation** | **P**  **Pearson** | **Pneumonia (n=11)** | **P MW** | **Pearson-**  **Correlation** | **P**  **Pearson** | **Cardiac arrhythmia (n=4)** | **P**  **MW** | **Pearson-**  **Correlation** | **P**  **Pearson** | **SOFA Score increase ≥2 (n=17)** | **P MW** | **Pearson- Correlation** | **P**  **Pearson** |
| --- | --- | --- | --- | --- | --- | --- | --- | --- | --- | --- | --- | --- | --- | --- | --- | --- | --- | --- |
| **DPP3 ng/ml** | Baseline | 37.91 $\pm$ 37.01 | 40.76 $\pm$ 23,76 | 0.35 | 0.016 | 0.95 | 44.89 $\pm$ 37.75 | **0.02*** | -0.05 | 0.83 | 69.22 $\pm-$ | 0.31 | 0.20 | 0.46 | 41.01 $\pm$ 37.81 | 0.21 | 0.21 | 0.4 |
|  | 24 h | 43.92 $\pm$ 38.66 | 76.95 $\pm-$ | **0.01*** | 0.19 | 0.42 | 52.78 $\pm$ 34.19 | 0.57 | 0.47 | **0.039** | 44.98 $\pm45.22$ | 0.65 | -0.006 | 0.97 | 49.87 $\pm$ 41.16 | 0.42 | -0.08 | 0.96 |
|  | 72 h | 32.30 $\pm$ 34.00 | 150.1 $\pm-$ | 0.10 | 0.81 | **<0.01** | 40.59 $\pm$ 54.59 | 0.83 | 0.23 | 0.32 | 85.53 $\pm91.32$ | 0.23 | 0.53 | **0.019** | 38.72 $\pm$ 38.75 | 0.18 | 0.56 | **0.01** |
|  | 120 h | 36.14 $\pm$ 34.34 | 39.07 $\pm$35.11 | 0.63 | 0.028 | 0.91 | 52.35 $\pm$ 43.20 | **0.07** | 0.42 | 0.072 | 44.04. $\pm31.68$ | 0.25 | 0.091 | 0.71 | 46.87 $\pm38.77$ | **0.006*** | 0.40 | **0.08** |
|  | 168 h | 30.70 $\pm$ 24.39 | 34.81$\pm$32,45 | 0.87 | 0.06 | 0.78 | 34.93 $\pm$ 26.88 | 0.59 | 0.21 | 0.38 | 44.06 $\pm13.48$ | 0.29 | 0.17 | 0.47 | 39.84 $\pm$ 26.38 | **0.09*** | 0.29 | 0.23 |
| **CRP mg/ml** | Baseline | 6.18 $\pm$ 3.16 | 4.7 $\pm$ 1.45 | 0.25 | -0.31 | 0.26 | 139.79 $\pm$ 104.48 | 0.12 | 0.51 | 0.026 | 6.90 $\pm$ - | 0.7 | 0.050 | 0.86 | 6.32 $\pm3.21$ | - | 0.93 | 0.74 |
|  | 24 h | 111.24 $\pm$ 69.08 | 128.71 $\pm-$ | 0.73 | 0.05 | 0.83 | 215.09 $\pm$ 122.15 | 0.23 | 0.59 | **0.007** | 134.13 $\pm7.66$ | 0.57 | 0.10 | 0.67 | 113.69 $\pm70.07$ | 1.0 | -0.29 | 0.22 |
|  | 72 h | 165.86 $\pm$ 70.23 | 330.18 $\pm-$ | 0.30 | 0.55 | **0.015** | 228.34 $\pm$ 83.66 | **0.03*** | 0.42 | 0.07 | 277.51 $\pm74.49$ | 0.07 | 0.54 | **0.016** | 166.62 $\pm72.07$ | 0.83 | 0.34 | 0.15 |
|  | 120 h | 142.32 $\pm$ 88.05 | 211.68 $\pm$47.61 | **0.02*** | 0.32 | 0.172 | 163.89 $\pm$ 109.75 | 0.54 | 0.32 | 0.18 | 177.03. $\pm97.64$ | 0.48 | 0.15 | 0.52 | 146.06 $\pm89.30$ | 0.57 | 0.07 | 0.73 |
|  | 168 h | 159.22 $\pm$ 98.04 | 210.63 $\pm100.66$ | 0.41 | 0.24 | 0.31 | 194.94 $\pm$ 97.85 | 0.27 | 0.32 | 0.17 | 168,23 $\pm6.13$ | 1.0 | 0.03 | 0.89 | 159.22 $\pm98.04$ | 0.65 | 0.36 | 0.12 |
| **Leukocytes billion/ml** | Baseline | 9.28 $\pm$ 2.81 | 8.13 $\pm1.93$ | 0.35 | -0.17 | 0.45 | 8.13 $\pm$1.93 | 0.30 | -0.17 | 0.45 | 11.91 $\pm$ - | 0.52 | 0.21 | 0.37 | 9.32 $\pm$ 2.88 | 1.0 | -0.28 | 0.21 |
|  | 24 h | 11.00 $\pm$ 2.62 | 13.69 $\pm$ | 0.3 | 0.24 | 0.30 | 11.00 $\pm$ 2.62 | **0.05*** | 0.38 | 0.09 | 10.41 $\pm3.07$ | 0.84 | -0.11 | 0.65 | $11.39 \pm2.55$ | 0.11 | 0.41 | 0.07 |
|  | 72 h | 10.27 $\pm$ 3.53 | 19.77 $\pm$ | 0.1 | 0.63 | **0.03** | 10.21 $\pm$ 3.53 | 0.81 | 0.23 | 0.92 | 13.52 $\pm7.4$ | 0.49 | 0.31 | 0.19 | $10.88\pm3.98$ | 0.23 | 0.44 | **0.05** |
|  | 120 h | 10.08 $\pm$ 3.83 | 11.97 $\pm$ 4.94 | 0.61 | 0.21 | 0.37 | 10.08 $\pm$ 3.83 | 0.38 | 0.10 | 0.67 | 13.45 $\pm7.2$ | 0.35 | 0.37 | 0.11 | $11.05\pm3.94$ | 0.06 | 0.32 | 0.16 |
|  | 168 h | 11.08 $\pm$ 4.64 | 12.34 $\pm$ 3.15 | 0.30 | 0.11 | 0.62 | 11.08 $\pm$ 4.64 | 0.76 | -0.1 | 0.65 | 19.76 $\pm6.7$ | **0.04*** | 0.63 | **0.03** | $11.39 \pm5.02$ | 0.67 | 0.14 | 0.53 |
| **PCT** $\boldsymbol{\mu}$**g/ml** | Baseline | 0.02 $\pm$ 0.03 | 0.2 $\pm0.04$ | 0.90 | -0.02 | 0.93 | 0.02 $\pm$ 0.03 | 0.77 | 0.15 | 0.51 | 0.02 $\pm$ - | 0.44 | -0.16 | 0.49 | 0.026 $\pm$ 0.03 | 0.44 | 0.38 | 0.97 |
|  | 24 h | 0.39 $\pm$ 0.57 | 1.47 $\pm-$ | 0.16 | 0.44 | **0.05** | 0.97 $\pm$ 0.7 | 0.11 | 0.34 | 0.14 | 1.47 $\pm$ - | 0.16 | 0.44 | **0.05** | 0.41 $\pm$ 0.61 | 0.70 | 0.17 | 0.46 |
|  | 72 h | 0.29$\pm$ 0.48 | 0.71 $\pm-$ | 0.29 | 0.19 | 0.40 | 0.37 $\pm$ 0.45 | 1.0 | 0.12 | 0.60 | 0.82 $\pm$ 0.15 | 0.06 | 0.36 | 0.11 | 0.36 $\pm$ 0.56 | 1.00 | 0.28 | 0.22 |
|  | 120 h | 0.28 $\pm$ 0.35 | 0.61 $\pm0.49$ | 0.08 | 0.41 | 0.06 | 0.31 $\pm$ 0.37 | 0.76 | 0.08 | 0.71 | 0.92 $\pm$0.20 | **0.01*** | 0.78 | **<0.01** | 0.27 $\pm$ 0.37 | 0.59 | 0.4 | 0.07 |
|  | 168 h | 0.25 $\pm$ 0.49 | 0.93$\pm1.13$ | 0.28 | 0.60 | **0.05** | 0.40 $\pm0.63$ | 0.09 | 0.35 | 0.12 | 0.21 $\pm$ 0.36 | 0.52 | -0.03 | 0.87 | 0.34 $\pm$ 0.62 | 1.00 | 0.50 | **0.02** |

**Supplementary Data Table 1: Comparative analysis of DPP3, CRP, leukocytes, and PCT levels across multiple timepoints in patients with postoperative complications. Statistical significance assessed with Mann-Whitney U *(MW)* *and* Bonferroni-adjusted (*)*,* and Pearson Correlation.**
